# Cerebrospinal fluid from Alzheimer’s disease patients promotes tau aggregation in transgenic mice

**DOI:** 10.1186/s40478-019-0725-3

**Published:** 2019-05-07

**Authors:** Zhiva Skachokova, Alfonso Martinisi, Martin Flach, Frederik Sprenger, Yvonne Naegelin, Viviane Steiner-Monard, Marc Sollberger, Andreas U. Monsch, Michel Goedert, Markus Tolnay, David T. Winkler

**Affiliations:** 1 Institute of Medical Genetics and Pathology, University Hospital Basel, Petersgraben 4, CH-4031 Basel, Switzerland; 2 Department of Neurology, University Hospital Basel, Petersgraben 4, CH-4031 Basel, Switzerland; 3 Memory Clinic, University Center for Medicine of Aging, Felix Platter Hospital & University of Basel, Burgfelderstrasse 101, CH-4002 Basel, Switzerland; 4 MRC, Laboratory of Molecular Biology, Francis Crick Avenue, Cambridge, CB2 0QH UK; 5 Neurology, Medical University Clinic, Kantonsspital Baselland, Rheinstrasse 26, 4410 Liestal, Switzerland

**Keywords:** Alzheimer’s disease, Neurodegeneration, Tau, Cerebrospinal fluid, Seeding, Prion, Transgenic mouse models

## Abstract

Tau is a microtubule stabilizing protein that forms aggregates in Alzheimer’s disease (AD). Tau derived from AD patients’ brains induces tau aggregation in a prion-like manner when injected into susceptible mouse models.

Here we investigated whether cerebrospinal fluid (CSF) collected from patients diagnosed with probable AD or mild cognitive impairment (MCI) likely due to AD harbors a prion-like tau seeding potential. CSF was injected intrahippocampally into young P301S tau transgenic mice. CSF obtained from AD or MCI patients increased hippocampal tau hyperphosphorylation and tau tangle formation in these mice at 4 months post-seeding. Tau pathology was also accentuated in the contralateral hippocampus, and in anterior and posterior directions, indicative of spreading.

We provide first evidence for in vivo prion-like properties of AD patients’ CSF, accelerating tau pathology in susceptible tau transgenic mice. This demonstrates that biologically active tau seeds reach the CSF compartment in AD. Further studies may help to evaluate strain specific properties of CSF derived tau bioseeds, and to assess their diagnostic potential.

**Electronic supplementary material:**

The online version of this article (10.1186/s40478-019-0725-3) contains supplementary material, which is available to authorized users.

## Introduction

Tau is a soluble protein that promotes microtubule assembly in neuronal cells. However, in pathological conditions, it becomes hyperphosphorylated and forms intracellular aggregates. This is characteristic of Alzheimer’s disease (AD), but also of other neurodegenerative disorders known as tauopathies, including cases of frontotemporal dementia (FTD), corticobasal degeneration (CBD), and progressive supranuclear palsy (PSP) [36]. In AD, tau aggregation propagates from one brain region to another in specific patterns [6, 18, 20, 21, 23]. It has been shown that tau pathology can be seeded in a prion-like manner, as the injection of brain extracts from tau transgenic donor mice or tauopathy patients into tau transgenic host mice causes tau aggregation in the inoculated mice [1, 8, 9, 24, 26, 27].

Pathologically modified tau forms are present in the extracellular space and can transfer between cells in the brain [10, 17, 40]. Phosphorylated tau can be secreted via exosomal release, and reach the cerebrospinal fluid (CSF) [32]. CSF tau levels are currently being used for the clinical diagnosis of AD in conjunction with cognitive tests, brain imaging and CSF amyloid-β (Aβ) level measurements [34]. Increased tau levels in the CSF correlate best with cognitive decline in AD patients.

Information on the prion-like properties of Aβ and tau from CSF in AD is limited. It has been shown that CSF amyloid-β does not exhibit prion-like properties in vivo [16, 35]. Recently, two studies reported a seeding-like potential of CSF-derived tau in vitro [31, 38]. It is however not known, whether tau-comprising CSF harbors seeding potential in vivo*.*

Here, we studied the in vivo seeding capacity of CSF derived from AD patients upon inoculation into P301S tau transgenic mice. These animals develop tau hyperphosphorylation and neurofibrillary tangles (NFT) when ageing. We collected CSF from patients diagnosed with probable AD or mild cognitive impairment (MCI) likely due to AD, and injected it into young P301S tau mice. As a result, we observed a significant increase in neurons positive for hyperphosphorylated tau, and an accelerated formation of Gallyas-Braak positive aggregates in the hippocampus, when compared to mice inoculated with CSF derived from elderly control patients. In parallel, there were signs of spreading of tau pathology along anatomical networks, in the form of hyperphosphorylated tau-positive neurons and dot-like structures, comparable to findings when seeding with brain-derived tau in P301S tau mice [1]. These findings provide first in vivo evidence for the presence of bioactive tau seeds in CSF.

## Materials and methods

### Mice

Homozygous human P301S mutant tau transgenic mice (P301S tau mice) [3] were used as host mice for all seeding experiments. Animal experiments were performed in compliance with protocols approved by the official local Committee for Animal Care and Animal Use of the Canton of Basel (License Nr. BS2471).

### CSF collection and patient characteristics

In order to obtain large volumes of CSF from AD and control patients, allowing subsequent concentration, CSF was collected within a clinical trial set up in collaboration with the Memory Clinic, University Center for Medicine of Aging, Basel. The trial was approved by the local Ethics Commission of the Canton of Basel, and recruitment was limited to patients with a Mini Mental State (MMS) Score [14] > 19/30. All patients examined for memory disturbances and potential neurodegenerative disorders at the Memory Clinic were consecutively allowed to enter the study. Up to 15 ml of CSF per patient were collected by lumbar puncture from a total of 23 subjects after obtaining written informed consent of the patient and a care-giver. Freshly collected CSF was spun at 3000 rpm for 30 min to remove cell debris. The supernatants were collected and frozen at − 80 °C. Clinical diagnosis was based on multimodal examinations including neuropsychological assessments, brain MRI imaging, CSF tau and Aβ level measurements. Brain perfusion SPECT imaging data was available on a subset of the patients.

### CSF processing

In order to obtain reasonable tau levels in small volumes that could be injected into mouse brains, CSF samples were concentrated [35]. In brief, 10 ml CSF per patient, was lyophilized (− 80 °C, 0.01 mbar vacuum pressure), reconstituted in sterile H_2_O and dialyzed (using Float-A-Lyzer G2, 1kD, Spectrum Labs) to reduce the salt load, re-lyophilized, and finally reconstituted in about 10 μl H_2_O (Additional file 1: Figure S1). The final concentration of tau was measured by ELISA.

### Elisa

CSF levels of tau, phospho-tau and Aβ, were measured by commercial kits “Innotest hTAU AG”, “Innotest PHOSPHO-TAU”, and “Innotest β-Amyloid (1-42)” according to the manufacturer’s guidelines (Innogenetics/Fujirebio Europe N.V., Belgium) [4, 37].

### Stereotaxic surgery

Three month-old P301S tau mice were anaesthetized with a mixture of ketamine (10 mg/kg) and xylazine (20 mg/kg) and placed on a heating pad to maintain body temperature during surgery. Mice were injected in the right hippocampus (A/P, − 2.5 mm from bregma; L, − 2.0 mm; D/V, − 1.8 mm) using a Hamilton syringe. Each mouse received a unilateral stereotaxic injection of 5 μl concentrated CSF, at a speed of 1.25 μl/min. Following the injection, the needle was kept in place for an additional 3 min. The surgical area was cleaned with saline and the incision sutured. Mice were monitored until recovery from anaesthesia, provided analgetic medication for up to 48 h post-surgery, and checked regularly following surgery.

### Immunohistochemical analysis

Four months post-inoculation, the mice were deeply anaesthetized with pentobarbital (150 mg/kg) and perfused with 20 ml cold PBS, followed by 20 ml 4% paraformaldehyde in PBS. The brains were dissected and post-fixed overnight. Following paraffin embedding, 5 μm thick coronal sections were prepared. Sections were silver-impregnated following the method of Gallyas-Braak to visualize filamentous tau pathology [7, 19]. Hematoxylin and eosin staining (H&E) was performed for morphological analysis. For tau immunohistochemistry AT8 (1:1000, Thermo Scientific), and AT100 (1:1000, Thermo Scientific) were used [29]. Secondary antibodies were from Vector Laboratories, Burlingame, CA (Vectastain ABC kit).

For quantification, 3 brain tissue sections were analyzed at levels comprising the injection site (from − 2 to − 3 μm from bregma), and the number of Gallyas-Braak silver stained inclusions, AT8 and AT100 positive neurons (entire hippocampus) and dot-like structures (dentate gyrus (DG), fimbria) was counted on 4X, 10X, or 20X magnified images, taken with an Olympus BX43 Upright Microscope (Life Science). For a few stainings, only 2 sections next to the indicated bregma levels were well preserved and usable for quantification. Bregma levels were determined by visual comparison with the Mouse Brain Atlas (Franklin and Paxinos, 2007). The quantifications and the measurements of the area of the respective anatomical regions were performed by the Cell counter plugin in ImageJ (imagej.net). Brain images that were used and modified are from the Mouse Brain Atlas (Franklin and Paxinos, Elsevier 2007).

AT8 positive neurons were furthermore semi-quantitatively assessed at various bregma levels in CA1, CA3, and dentate gyrus (DG) regions, and an average score was obtained. For the heatmap (Fig. 2), average tau pathology per bregma level was calculated and color-graded.

### Statistical analysis

In order to estimate the effects of AD CSF tau seeds versus control seeds, mean values of counts/area were calculated, and *p*-values were obtained by t-tests. Data was collected from 3 to 6 seeded mice per group, depending on the stainings, as indicated in Additional file 1: Table S1. *P*-values are interpreted exploratory and are not adjusted for multiple comparisons. In the results section and the figures, mean values and standard deviations are indicated. A *p*-value < 0.05 was considered as significant. A comprehensive overview on the statistical data is provided in Additional file 1: Table S1.

### Data sharing

All relevant clinical data on the patients included in this exploratory translational study is provided in Table 1. Additional individual de-identified patient data will be shared by the corresponding author.

**Table 1:** Clinical, imaging, and laboratory findings of the patients selected for the preparation of CSF seeds (CTR CSF: control patients’ group, AD CSF: Alzheimer’s disease group). Bold letters indicate pathological values of CSF tau, p-tau or Aβ. NIA-AA criteria: National Institute on Aging-Alzheimer’s Association workgroups on diagnostic guidelines for Alzheimer’s disease. Criteria abbreviation: BP = “Biomarker probability of AD etiology” [2, 25]

patient number	group	diagnosis NIA-AA criteria	alternative clinical diagnosis	age	MMS	Brain MRI	SPECT	CSF tau (pg/ml)	CSF p-tau (pg/ml)	CSF Aβ (pg/ml)
1	CTR	Dementia unlikely to be due to AD	vascular encephalopathy	80	26	vascular encephalopathy	no signs of AD or FTD, compatible with vascular encephalopathy	500	**78**	544
2	CTR	Dementia unlikely to be due to AD	neurocognitive deficits associated with progressive pancreas carcinoma and tumorectomy	73	25	mild vascular microangiopathy	–	206	50	580
3	CTR	MCI - unlikely to be due to AD	early Wernicke–Korsakoff syndrome	61	30	compatible with Wernicke-Korsakoff syndrome	compatible with Wernicke-Korsakoff syndrome	162	43	928
4	CTR	MCI - unlikely to be due to AD	depression	57	28	normal	–	360	53	1133
1	AD	Probable AD with high BP	–	73	25	hippocampal atrophy, no vascular lesions	–	**840**	**105**	**366**
2	AD	Probable AD with high BP	–	61	24	temporal brain atrophy	Left parietal decreased glucose metabolism compatible with early AD	**500**	**92**	**318**
3	AD	MCI - core clinical data	–	79	28	mild hippocampal atrophy	–	**759**	**119**	516
4	AD	Probable AD with intermediate BP	–	71	23	left hippocampal atrophy, microangiopathy	–	308	**77**	**240**
5	AD	Probable AD with intermediate BP	–	74	21	hippocampal and global brain atrophy, mild microangiopathy	–	238	32	**402**

## Results

### CSF derived from AD patients accelerates cerebral tau pathology in P301S host mice

#### CSF collection: patients’ characteristics

CSF was prospectively from a total of 23 patients visiting an outpatient Memory Clinic, in the setting of an explorative clinical trial. Up to 15 ml of CSF were collected per patient without any relevant side effects. Clinical, imaging, and laboratory data of all patients were assessed. For experimental inoculation into transgenic mice, we selected CSF samples derived from the patients with the highest (“Alzheimer’s disease patients”: AD group, *n* = 5) and the lowest (“control”-patients: CTR group, *n* = 4) probability of AD-related cognitive decline. AD CSF samples were included from 2 patients fulfilling the actual criteria for “Probable AD dementia with high biomarker probability of AD etiology”, two patients with “Probable AD dementia with intermediate biomarker probability of AD etiology” [25], as well as one patient with “MCI - core clinical data” [2], the latter with high evidence for neuronal injury in the CSF analysis [2]. The 4 patients attributed to the CTR CSF group fulfilled the criteria for “Dementia-unlikely to be due to AD” (*n* = 2) or “MCI-unlikely due to AD” (n = 2), without biomarker evidence for AD [2, 25]. Detailed patients’ characteristics are provided in Table 1. Cognitive decline of control patients was clinically attributed to non-neurodegenerative causes (e.g. vascular dementia or alcohol abuse). Mean age (CTR 67,8y/AD 71,6y, *p* = 0,52) and MMS Score (27,3/24,2, *p* = 0.10) did not significantly differ between the two groups, although there was a trend to a higher MMS in the patients attributed to the control patients’ group.

Given the physiologically low tau concentrations in CSF in the ng/ml range, the samples were concentrated as previously described (Additional file 1: Figure S1) [16, 35]. After concentration, no significant difference of the total tau levels between the two groups was measured by ELISA (156 ng/ml / 204,6 ng/ml, *P* = 0.65, Additional file 1: Figure S2).

#### Hippocampal neuronal tau pathology provoked by AD-patients’ CSF

**Fig. 1:**
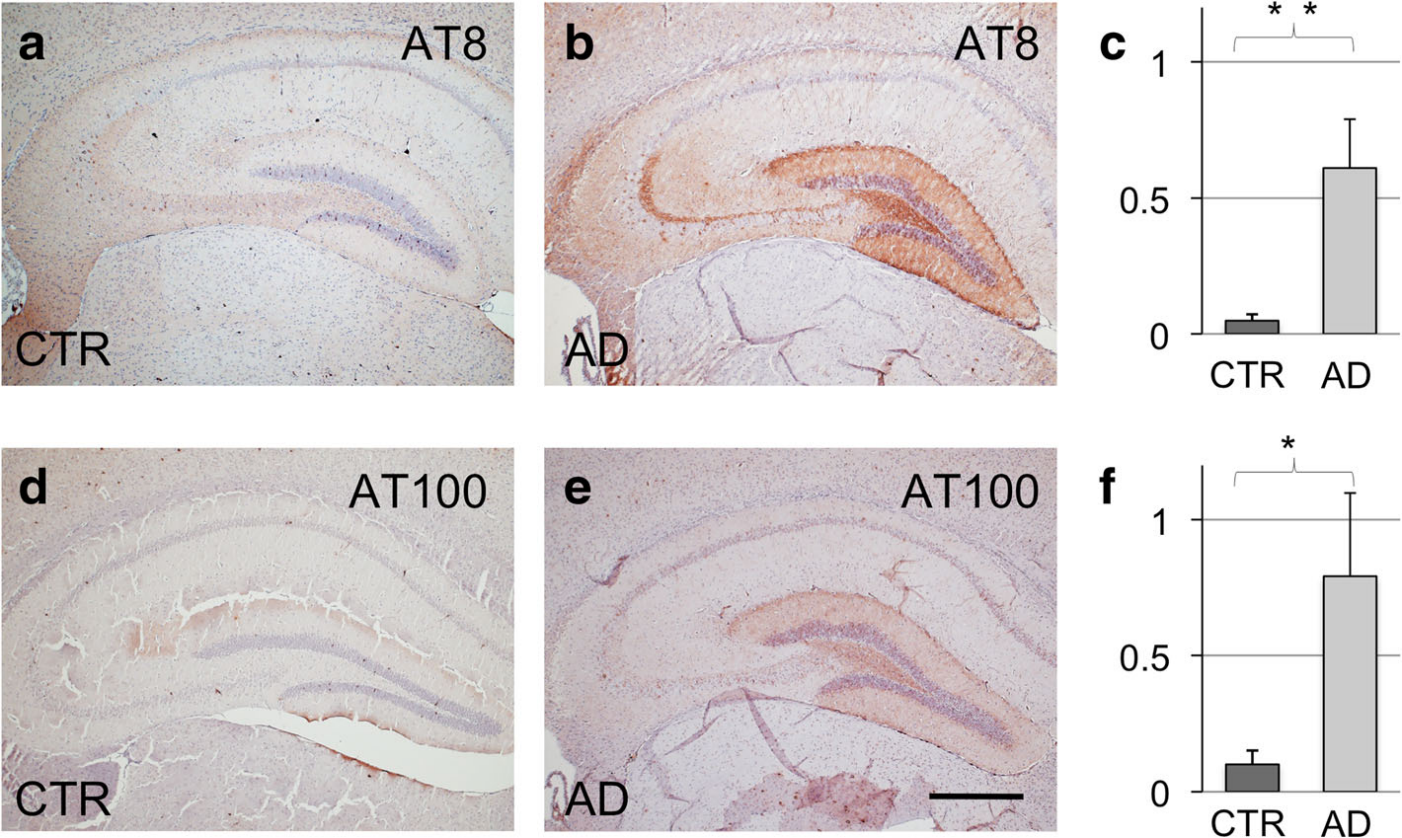
Immunohistochemistry for AT8 and AT100 tau phosphorylation markers of 7 months old P301S transgenic mice, sacrificed 4 months after unilateral hippocampal inoculation with CSF derived from control patients (CTR) or AD patients (AD) (**a**, **d**; **b**, **e**). AT8 positive hippocampal neurons (**c**, *p* = 0,001) ipsilateral to the inoculation site; AT100 positive hippocampal neurons, ipsilateral (**f**, p = 0,0013). Numbers indicate neurons per area. Scale bar in e equals 500 μm, and applies to **a**, **b**, **d**, and **e**

Intrahippocampal injections of AD-patients’ CSF into young P301S tau mice resulted in an increased number of AT8 positive hippocampal neurons at the injection level, as compared to CTR CSF inoculated mice (Fig. 1a-c, mean neuron count per area CTR CSF/AD CSF: 0,049/0,611, *p* = 0,001, for comprehensive overview on the statistical data see Additional file 1: Table S1). Immunohistochemistry for AT100 also showed a significant increase in positive hippocampal neurons in the AD CSF injected hippocampus when compared to CTR CSF administered mice (Fig. 1e-f, CTR CSF/AD CSF: 0,101/0,729, *p* = 0,013, for a detailed view on the accentuated AT8 and AT100 pathology in the CA1 field see Additional file 1: Figure S3). Moreover, a trend to increased tau phosphorylation was noticeable also in the contralateral, non-injected hippocampus, both for AT8 as well as for AT100 hyperphosphorylated tau, when comparing the CTR group to the AD group (AT8: 0,050/0,393, p = 0,053, AT100: 0,164/0,626, p = 0,053).

**Fig. 2:**
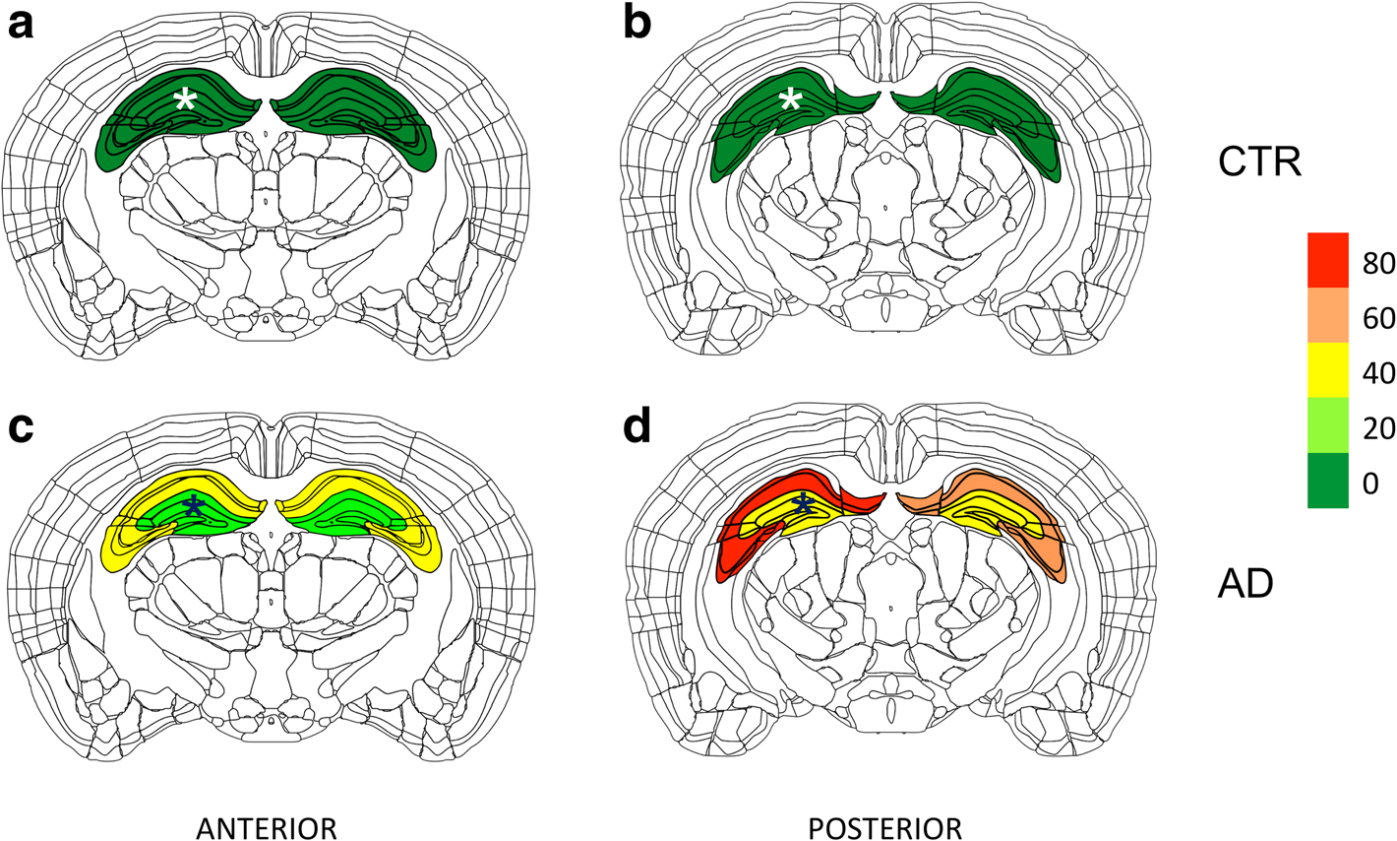
Heat map of a semiquantitative analysis of AT8 tau positive neurons anterior and posterior, as well as ipsilateral (asterisks) and contralateral to the injection site after CTR CSF and AD CSF seedings. Bregma levels 2–2.5/2–2.5 mm (**a**, **c**, anterior), and 2.5–3/2.5–3 mm (**b**, **d**, posterior)

Semi-quantitative analysis revealed more AT8 positive hippocampal neurons in AD CSF seeded mice anterior (from − 2 to − 2.5 mm) and posterior (from − 2.5 to -3 mm) to the injection site, compared to CTR CSF seeded mice, both, ipsi-, and contralateral to the injection site (Fig. 2). This is compatible with CSF-induced spreading of tau pathology throughout the hippocampus of inoculated mice along anatomically connected regions, similarly to previous findings when seeding with tau containing brain homogenates [1, 8, 33].

**Fig. 3:**
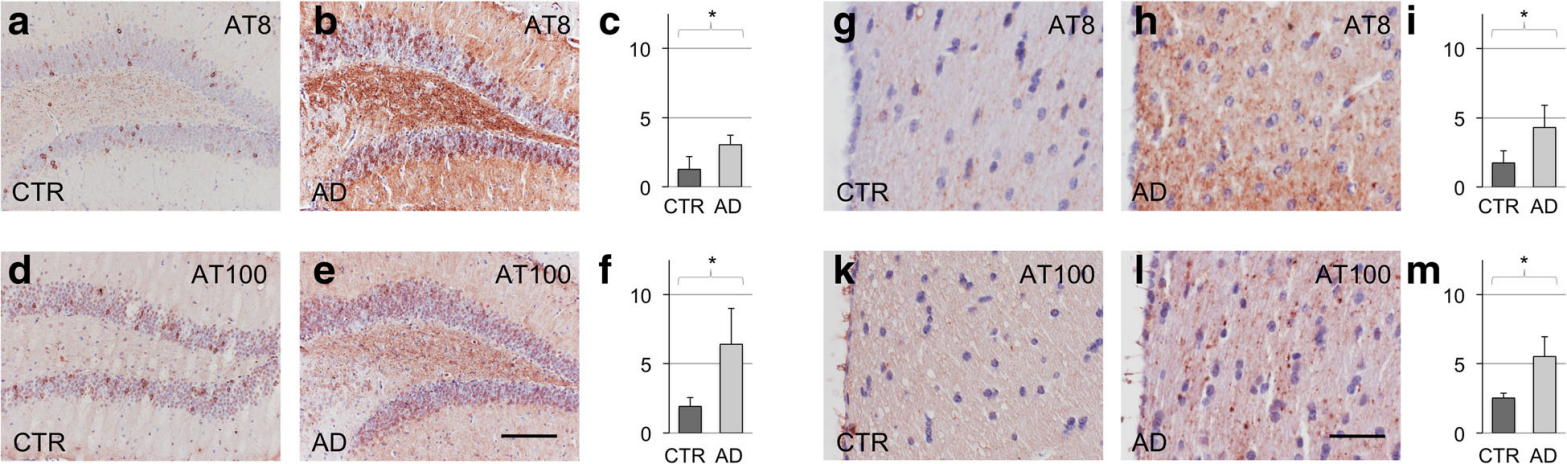
AT8 and AT100 immunohistochemistry after unilateral hippocampal seeding with CTR CSF (**a**, **g**; **d**, **k**) and AD CSF (**b**, **h**; **e**, **l**) into P301S mice. Accumulation of AT8 (**c**, *p* = 0.013) and AT 100 (**f**, *p* = 0.036) positive dot-like structures in the DG ipsilateral to the injection site. Increase in AT8 (**i**, *p* = 0.039) and AT 100 (**m**, *p* = 0.018) positive dot-like structures in the fimbria. Numbers indicate dot like structures per area. Scale bar in e equals 150 μm in **a**, **b**, **d** and **e**; scale bar in **l** equals 37,5 μm in **g**, **h**, **k**, and **l**

Previous seedings with brain homogenates in P301S mice resulted in a prominent increase in dot-like structures, attributable to axonal tau aggregates [1]. Hippocampal tissue from AD CSF seeded mice showed similar wide-spread dot-like structures, when stained for AT8 or AT100. Dot-like structures were most prominent in the dentate gyrus (DG) (Fig. 3a-f, Additional file 1: Figure S4a-d), as well as in the pathways of the fimbria (Fig. 3g-m). In CTR CSF seeded mice, some dot-like structures were also present, most prominently in the DG (Fig. 3a, d), and accentuated in the dorsal hippocampus, where they occur regularly in untreated aged P301S mice. When comparing CTR CSF to AD CSF inoculated mice, the increase in AT8 and AT100 positive dot-like structures in the DG was significant ipsilateral to the injection site (AT8: 1,265/3,045, *p* = 0,013 (Fig. 3a-c); AT100: 1,930/6,400, p = 0,036 (Fig. 3d-f)), and even contra-lateral to the injection site (AT8: 1,012/2,574, p = 0,031; AT100: 3,121/6,266, p = 0,047). Compatible with an induced spreading of tau aggregation, an increase of tau hyperphosphorylation was also noted in the fimbria, both for AT8 and AT100 positive granular structures (AT8: 1,736/4,310, p = 0,039 (Fig. 3g-i); AT100: 2539/5514, p = 0,018 (Fig. 3k-m)), while showing a similar trend on the contra-lateral side (AT8: 1,603/3,258, p = 0,121; AT100: 2,598/4,302, p = 0,053).

**Fig. 4:**
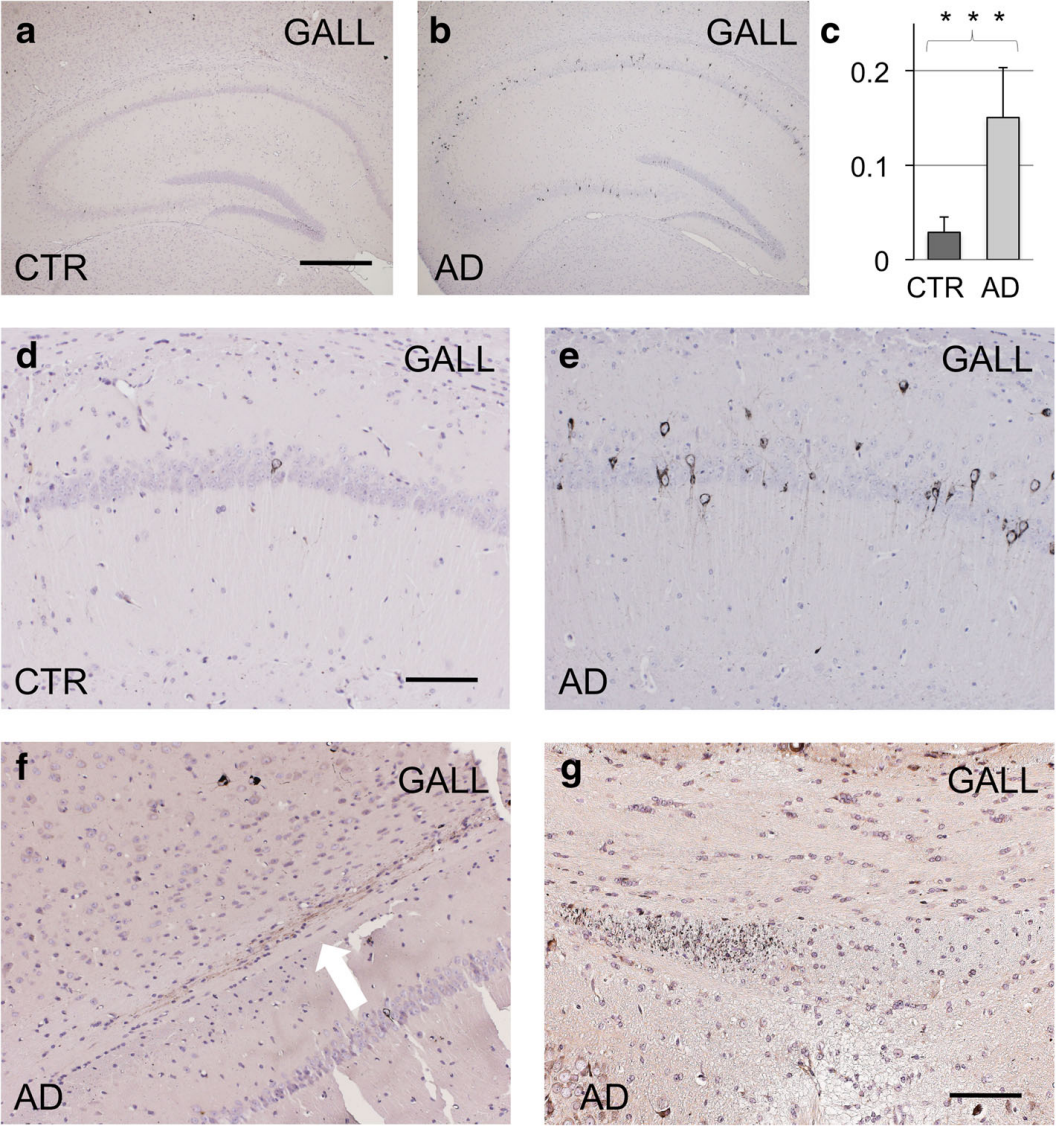
Gallyas-Braak stainings. Overview on the hippocampus (CTR CSF: **a**, AD CSF: **b**). Increase in Gallyas-Braak positive hippocampal neurons in AD CSF seeded mice compared to CTR CSF seedings (**c**, *p* = 0.0007). Gallyas positive neurons in the CA1 subfield (CTR CSF: d, AD CSF: **e**). Focal dot-like Gallyas-Braak positive structures in the ipsilateral alveus above the CA1 field after inoculation of CSF from patient #2 (**f**, arrow), and in the ipsilateral dorsal fornix after seeding with CSF from patient #1 (**g**). Numbers indicate neurons per area. Scale bar in a equals 500 μm in a and b; scale bar in d equals 100 μm and applies to d-f; scale bar in g equals 76 μm

Furthermore, Gallyas-Braak staining showed an increase in silver positive hippocampal neuronal inclusions in AD CSF seeded host mice, both ipsilateral (Fig. 4a-c, 0,029/0,151, p = 0,0007), as well as contralateral to the injection site (0,049/0,128, *p* = 0.014). This is compatible with the induction of later stage tau hyperphosphorylation and tau aggregation by the AD CSF tau seeds, as also visible on higher magnification views of the CA1 region (Fig. 4d, e). Only in a subset of mice, focal granular aggregates of Gallyas-Braak positive tau structures became visible. In a mouse seeded with CSF collected from patient #2, we noted a focal induction of Gallyas-Braak positive grains in the ipsilateral alveus above the CA1 field (Fig. 4f), while in a mouse inoculated with CSF derived from patient #1, prominently focal granular tau structures appeared in the dorsal fornix, strictly limited to the inoculated side (Fig. 4g). The latter finding was reminiscent of previous observations of focal tau aggregation in the dorsal fornix following injection of human brain extracts derived from tauopathy patients [5].

## Discussion

Here we demonstrate the in vivo tau seeding effects of cerebrospinal fluid (CSF) derived from patients suffering from AD dementia or mild cognitive impairment (MCI) likely due to AD. CSF containing hyperphosphorylated tau species accelerated tau pathology after intrahippocampal inoculation into P301S tau transgenic host mice. These findings corroborate two recent reports of in vitro seeding-like activity of CSF tau [31, 38], providing now first evidence for biological seeding activity of patients’ derived CSF tau in susceptible host mice.

CSF collected from patients with a high probability of AD or related MCI was used, and compared with CSF derived from age-matched patients with diagnosis other than AD. Inoculation of CSF collected from AD and MCI patients resulted in a significant increase of markers of tau pathology in the hippocampus of P301S tau host mice. Advanced tau pathology was also notable in the contralateral hippocampus, and in anterior and posterior directions. This is comparable to the spreading we and others have described after seeding with brain extracts of tau transgenic mice or tauopathy patients [1, 5, 8, 9, 22].

Besides an overall increase in tau inclusion positive hippocampal neurons, injection of concentrated AD patients’ CSF into young P301S tau mice resulted in focal granular, dot-like tau accumulation particularly in the fimbria and the dentate gyrus (DG) in a subset of the inoculated mice. This granular pathology is very similar to earlier findings in tau transgenic mice seeded with brain extracts from a patient who suffered from corticobasal degeneration [5]. Punctate Gallyas-Braak silver staining positive structures indicative of axonal tau aggregates accumulated in the alveus and the dorsal fornix close to the injection site in two AD CSF inoculated mice, reminiscent of a similar pathology induced in the fornix of P301S tau mice seeded with P301S tau mouse donor brain extracts [1]. These characteristic focal Gallyas-Braak positive, axonal dot-like structures were visible in a subset of AD CSF inoculated host mice. Seeding patterns might differ due to varieties in the tau strains involved in the donor patients. It has been shown that brain samples from different tauopathy patients may accelerate tau aggregation variably in different structures or cell types, and also propagate in distinctive ways, indicating the presence of different tau strains [5]. Recently, different ultrastructural polymorphs of patients’ brain-derived tau strains have been identified by cryo-EM [12, 13].

Our findings suggest that in AD patients, pathological tau species with biological seeding competence drain from the brain interstitial fluid to the CSF compartment. There, they retain their potential to evoke seed-like effects in vivo, similarly as previously reported for brain parenchymal tau species.

The here observed tau seeding competence of AD patients’ CSF contrasts our earlier findings in seeding experiments with Aβ. There, even very long seeding times after inoculation of concentrated human or murine Aβ comprising CSF into APP transgenic host mice failed to provide evidence for the presence of bioactive Aβ seeds in the CSF compartment [16, 35]. The here described biological tau seeding activity of CSF is however not entirely surprising. In contrast to Aβ that aggregates broadly in the brain parenchyma and the perivascular space, tau is readily drained towards the CSF compartment. Opposite to Aβ, tau levels increase in the CSF with the progression of AD [28].

Furthermore, our evidence for in vivo seeding activity of CSF tau follows recent findings on the ability of CSF tau to induce seeding-like conformational changes in vitro [31, 38]. Ventricular CSF samples collected post mortem at autopsies of AD patients have been inducing tau aggregation in HEK293 cells expressing a P301S tau repeat domain [38]. CSF tau derived from Pick’s disease patients has furthermore been found to induce real-time quaking conversion (RT-QuIC) of a 3 repeat tau fragment [31]. In vitro seeding activity in cellular systems, or prion-like substrate conversion capacity by RT-QuIC, has furthermore recently been demonstrated for CSF in the context of other neurodegenerative proteinopathies, including sporadic Creutzfeldt Jakob disease [15] and Huntington’s disease [39].

The presence of bioactive tau seeds in the CSF of AD patients suggests a diagnostic use of the aggregation induction capacity of CSF in tauopathies. In contrast to brain tissue, CSF can easily be collected from patients with a low rate of side effects [30]. The simple analysis of CSF tau levels is an established part of the diagnostic criteria for AD, but it lacks yet high specificity and is unable to provide prognosis at early or even preclinical disease stages [11, 28]. The latter would however be important also for the development of novel therapies. Our detection of bioactive tau seeds in CSF of AD patients augurs well for the development of novel diagnostic tools that are based on the specific conformational properties and the bioactivity of tau.

The biological in vivo seeding capacity of AD patients’ CSF that we describe calls for considerations on the biosafety of operators when handling CSF samples. Compared to previous seedings with brain tissues [8], the seeding effects described here after inoculation of concentrated CSF into a tau overexpressing murine host system are relatively mild. We therefore consider the seeding potential of AD tau in CSF rather low. We however did not compare the seeding capacity of AD CSF to proportionately diluted AD brain lysates, so that a direct comparison of the seeding potential of CSF to brain-derived tau remains yet to be done. The host mice used in the present study furthermore express a human P301S mutant tau form which is particularly prone to aggregation. The present findings therefore can’t be directly translated into the setting where people with normal wild-type tau would be exposed to AD patients’ tau samples. Altogether, the seeding potential of CSF might be of limited relevance in a routine laboratory or clinical setting when adhering to standard safety procedures for the handling of potentially infectious bio-samples.

This study is subject to some limitations. Due to the large amounts of CSF needed, CSF had to be collected consecutively from donating patients. This resulted in a limited number of patients’ samples attributable to either the AD- or the control-group, similar to the previous in vitro study done in HEK293 cells [38]. As tau seeding is a time and concentration dependent process [9], we concentrated the CSF samples before the intracerebral inoculations. We can’t therefore exclude, that the seeding potential of tau was increased by the concentration process itself. The absence of any seeding activity in our previous experiments with concentrated CSF-Aβ [16, 35], as well as the significant differences between the CSF tau derived from AD patients versus the controls, however speak against a relevant influence of the concentrating procedure. Given the short life span of homozygous P301S tau mice, the maximum seeding time was limited to 4 months. We therefore can’t exclude that a more robust seeding response would become apparent at later time points post-seeding.

The exact nature of bioactive tau seeds has yet remained unknown. The need of large amounts of CSF for seedings in the present study has precluded any detailed analysis of the bioactive seeds. In their post mortal CSF samples, Takeda et al. found high molecular weight tau (HMW) species, and considered them to be the bioactive species in vitro [38]. Future studies will be needed to analyze the structural nature of CSF tau bioseeds.

In conclusion, we here provide first in vivo evidence for the presence of bioactive tau aggregation accelerating seeds in CSF of AD patients. Further studies will be needed to elaborate whether CSF derived bioactive tau seeds might be of use as a marker of AD, as well as to study mechanistic aspects of potentially strain specific patterns of CSF bioseeds in different tauopathies.

## Additional file

Additional file 1: **Figure S1.** CSF was collected from patients and controls by a lumbar puncture, obtaining about 10 ml on average. From those, 8 ml were used for subsequent lyophilization, dialysis, and re-lyophilization, and finally dissolved in 66 μl sterile water, with a theoretical concentration factor of 120x. **Figure S2.** Tau and phospho-tau (P-Tau, T231) concentration of the patients CSF used for seeding experiments, as measured by ELISA. There was no significant difference of the total tau levels between the AD and the CTR group, as measured by ELISA, before (0,530 ng/ml / 0,307 ng/ml, *p* = 0,183) and after concentration (204,6 ng/ml / 156 ng/ml, *p* = 0.646). **Figure S3.** Immunohistochemistry of the hippocampal CA1 field for AT8 and AT100 tau phosphorylation markers of 7 months old P301S transgenic mice, sacrificed 4 months after unilateral hippocampal inoculation with CSF derived from control patients (CTR) or AD patients (AD) (a-d). Scale bar in d equals 100 μm and applies to a-d. **Figure S4.** Immunohistochemistry of the dentate gyrus for AT8 and AT100 tau phosphorylation markers of 7 months old P301S transgenic mice, sacrificed 4 months after unilateral hippocampal inoculation with CSF derived from control patients (CTR) or AD patients (AD) (a-d). Scale bar in d equals 37,5 μm and applies to a-d. **Table S1.** Overview of the statistical data of unilateral intrahippocampal seedings with CSF derived from AD patients (AD) and control patients (CTR) into P301S mice. Abbreviations: HIPP: Hippocampal, DG: dentate gyrus, IPSI: ipsilateral to the intrahippocampal inoculation of the seed, CONTRA: contralateral to the intrahippocampal inoculation of the seed. (PDF 3360 kb)
