# Supplementary material for: Cerebrospinal fluid from Alzheimer’s disease patients promotes tau aggregation in transgenic mice
Source: Acta Neuropathol Commun. 2019 May 7;7:72. doi: 10.1186/s40478-019-0725-3 (PMC6503541; doi:10.1186/s40478-019-0725-3)
Supplement: Supplementary file 1 — Figure S1. CSF was collected from patients and controls by a lumbar puncture, obtaining about 10 ml on average. From those, 8 ml were used for subsequent lyophilization, dialysis, and re-lyophilization, and finally dissolved in 66 μl sterile water, with a theoretical concentration factor of 120x. Figure S2. Tau and phospho-tau (P-Tau, T231) concentration of the patients CSF used for seeding experiments, as measured by ELISA. There was no significant difference of the total tau levels between the AD and the CTR group, as measured by ELISA, before (0,530 ng/ml / 0,307 ng/ml, p = 0,183) and after concentration (204,6 ng/ml / 156 ng/ml, p = 0.646). Figure S3. Immunohistochemistry of the hippocampal CA1 field for AT8 and AT100 tau phosphorylation markers of 7 months old P301S transgenic mice, sacrificed 4 months after unilateral hippocampal inoculation with CSF derived from control patients (CTR) or AD patients (AD) (a-d). Scale bar in d equals 100 μm and applies to a-d. Figure S4. Immunohistochemistry of the dentate gyrus for AT8 and AT100 tau phosphorylation markers of 7 months old P301S transgenic mice, sacrificed 4 months after unilateral hippocampal inoculation with CSF derived from control patients (CTR) or AD patients (AD) (a-d). Scale bar in d equals 37,5 μm and applies to a-d. Table S1. Overview of the statistical data of unilateral intrahippocampal seedings with CSF derived from AD patients (AD) and control patients (CTR) into P301S mice. Abbreviations: HIPP: Hippocampal, DG: dentate gyrus, IPSI: ipsilateral to the intrahippocampal inoculation of the seed, CONTRA: contralateral to the intrahippocampal inoculation of the seed. (PDF 3360 kb) [file 40478_2019_725_MOESM1_ESM.pdf]

Supplemental Information

Supplemental Figures:

Figure S1: CSF processing

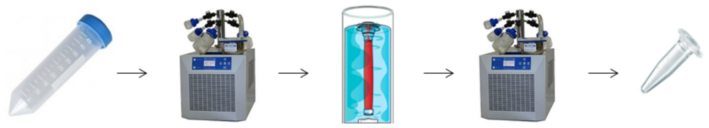

Figure S1: CSF was collected from patients and controls by a lumbar puncture, obtaining about 10ml on average. From those, 8ml were used for subsequent lyophilization, dialysis, and re-lyophilization, and finally dissolved in 66µl sterile water, with a theoretical concentration factor of 120x.

Figure S2: CSF tau concentrations

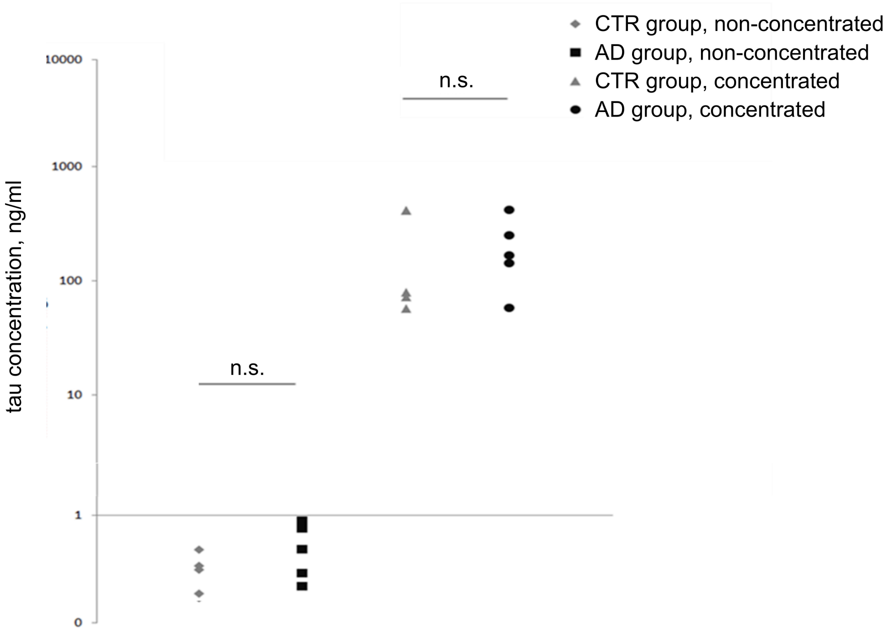

Figure S2: Tau and phospho-tau (P-Tau, T231) concentration of the patients CSF used for seeding experiments, as measured by ELISA. There was no significant difference of the total tau levels between the AD and the CTR group, as measured by ELISA, before (0,530 ng/ml / 0,307 ng/ml, p=0,183) and after concentration (204,6 ng/ml / 156 ng/ml, p=0.646).

Cerebrospinal fluid induced tau seeding
Skachokova Z. et al.

Figure S3: CA1 pathology

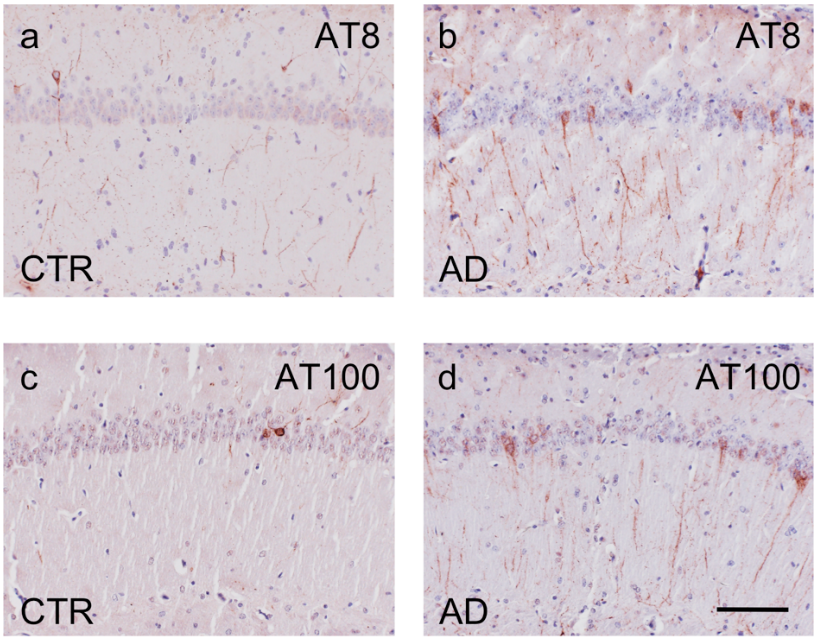

Figure S3: Immunohistochemistry of the hippocampal CA1 field for AT8 and AT100 tau phosphorylation markers of 7 months old P301S transgenic mice, sacrificed 4 months after unilateral hippocampal inoculation with CSF derived from control patients (CTR) or AD patients (AD) (a-d). Scale bar in d equals 100 μm and applies to a-d.

Figure S4: DG pathology

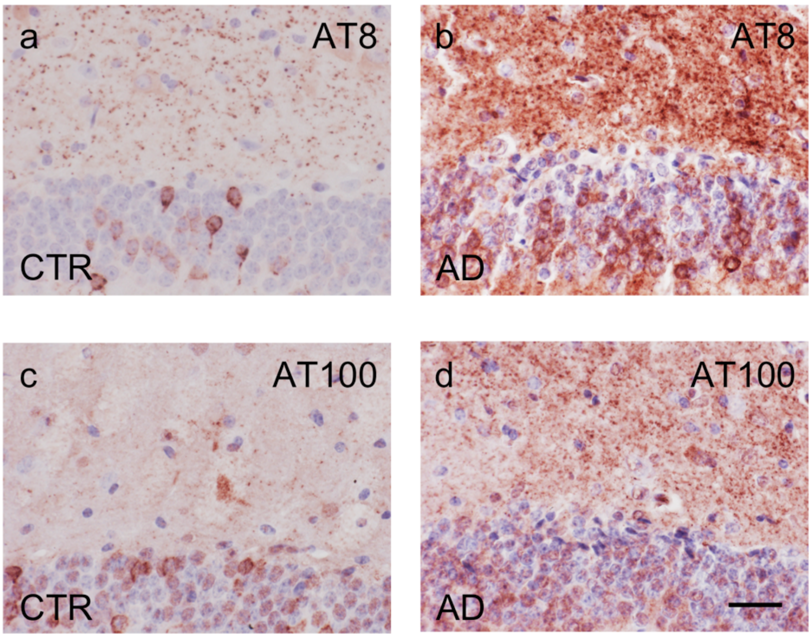

Figure S4: Immunohistochemistry of the dentate gyrus for AT8 and AT100 tau phosphorylation markers of 7 months old P301S transgenic mice, sacrificed 4 months after unilateral hippocampal inoculation with CSF derived from control patients (CTR) or AD patients (AD) (a-d). Scale bar in d equals 37,5 μm and applies to a-d.

Cerebrospinal fluid induced tau seeding
Skachokova Z. et al.

Supplemental Table:

Table S1: Statistical data CSF seedings

	AT8 HIPP neurons	AT100 HIPP neurons	Gallyas HIPP neurons	AT8 DG grains	AT100 DG grains	Gallyas DG grains	AT8 fimbria grains	AT100 fimbria grains	Gallyas fimbria grains
N per group (AD/CTR)	6/3	4/3	6/5	6/3	4/3	6/5	6/3	4/3	6/5
Mean IPSI (AD/CTR)	*0,611/ 0,049*	*0,792/ 0,101*	*0,151/ 0,029*	*3,045/ 1,265*	*6,400/ 1,930*	*1,660/ 1,616*	*4,310 /1,736*	*5,514/ 2,539*	*2,829/ 1,884*
Mean CONTRA (AD/CTR)	*0,393/ 0,050*	*0,626/ 0,164*	*0,128/ 0,049*	*2,574/ 1,012*	*6,266/ 3,121*	*2,379/ 2,232*	*3,258/ 1,603*	*4,302/ 2,598*	*2,619/ 1,744*
P value IPSI (AD vs CTR)	*0,001*	*0,013*	*0,0007*	*0,013*	*0,036*	*0,942*	*0,039*	*0,018*	*0,218*
P value CONTRA (AD vs CTR)	*0,053*	*0,053*	*0,014*	*0,031*	*0,047*	*0,884*	*0,121*	*0,053*	*0,309*

Table S1: Overview of the statistical data of unilateral intrahippocampal seedings with CSF derived from AD patients (AD) and control patients (CTR) into P301S mice. Abbreviations: HIPP: Hippocampal, DG: dentate gyrus, IPSI: ipsilateral to the intrahippocampal inoculation of the seed, CONTRA: contralateral to the intrahippocampal inoculation of the seed.
